# The Annalist

**Published:** 1848-10

**Authors:** 


### THE ANNALIST.

It is known to our readers that for some time past, a medical periodical with this title has been published, bi-weekly, in the city of New York. Our pages have borne witness of our appreciation of its character, by the numerous and valuable extracts from it which have from time to time appeared in the Examiner. In the last number, Sept. 15, we are sorry to find *The Farewell Address* of its learned and able editor, Dr. Roberts. After two years of labour and solicitude, he gives place to another, and the bantling, reared with so much midnight toil and at the sacrifice of so much social and domestic enjoyment, is henceforth to bear another's name. *Sic transit gloria mundi!* We have sometimes felt called upon to dissent from the views put forth in the Annalist, but much oftener have had occasion to commend, and often to admire, the high tone of the editor's remarks, when battling for the honour and welfare of our profession. To his successor, Dr. Davis, he leaves a bright example of editorial amenity.

